# Impact of hormone therapy side effects on health-related quality of life, distress, and well-being of breast cancer survivors

**DOI:** 10.1038/s41598-022-22971-x

**Published:** 2022-11-04

**Authors:** Y. Andreu, A. Soto-Rubio, M. Ramos-Campos, A. Escriche-Saura, M. Martínez, J. Gavilá

**Affiliations:** 1 Personality, Assessment and Psychological Treatments Department, Faculty of Psychology, University of Valencia, Valencia, Spain; 2 Asociación Española Contra el Cáncer (AECC), Valencia, Spain; 3 Department of Medical Oncology, Hospital Clínico Universitario de Valencia-Biomedical Research Institute INCLIVA, Valencia, Spain; 4 Medical Oncology Department, Fundación Instituto Valenciano de Oncología, Valencia, Spain

**Keywords:** Breast cancer, Quality of life, Hormonal therapies, Adverse effects, Risk factors, Human behaviour

## Abstract

To explore the modulatory role of Adjuvant Hormone Therapy (AHT) on health-related quality of life (QoL), subjective well-being and distress prevalence in Breast Cancer (BC) survivors, considering the survival phase. Cross-sectional study with control group. 616 BC survivors participated. Examination of interaction effect between AHT and time since end of primary treatment showed that many of the positive changes observed through the survival phases were experienced exclusively by survivors without AHT. When AHT was not prescribed, longer time elapsed was associated with a decrease in distress prevalence and an improvement in subjective well-being and QoL. It seems there is a turning point around the fifth year after finalization of primary treatment, from which the survivors without AHT significantly improve in several areas and those with AHT do so to a lesser extent. It is expected that the improvement in QoL throughout the different survival phases will have a significant impact on the adherence and maintenance of AHT and, consequently, the likelihood of survival. Thus, AHT side-effects should be routinely assessed by health care providers to gain accurate knowledge that allows improving the QoL of BC survivors.

## Introduction

Breast cancer (BC) is the world’s most prevalent cancer^1^. At the same time, the availability of increasingly effective therapies has contributed to improve the rates of survival in BC. According to the World Health Organization^1^, age-standardized BC mortality in high-income countries dropped by 40% between the 1980s and 2020, making BC survivors the largest group of cancer survivors^2^. In hormone receptor-positive BC patients, which account for the 70% of total BC^3^, one of the mainstays of treatment is the Adjuvant Hormone Therapy (AHT), which either suppresses hormone production (aromatase inhibitors such as Letrozole, Anastrozole and Exemestane) or interferes with hormone receptor signalling (Selective Estrogen Receptor Modulators such as Tamoxifen)^4^.

AHT reduces the risk of BC recurrence by 40% and mortality by a third^5^. The flip side of AHT is that it can also cause a wide range of side-effects that even though they are less severe in comparison to those of other treatments such as chemotherapy, it should be kept in mind that the treatment period with AHT is considerably longer, ranging from 5 to 10 years^6^.

Among the main physical health related side-effects are joint pain^7–12^, musculoskeletal symptoms such as pain throughout the whole body^8,9,13^, carpal tunnel syndrome^7^, cognitive side-effects such as difficulty concentrating and memory loss^14^, hot flushes and night sweats and the consequent loss of sleep^15–19^, general lack of energy^10,16,20,21^, and body image concerns due to weight gain^22^.

The presence and severity of the aforementioned AHT side-effects have been identified as barriers to treatment persistence and adherence^6,8,23–27^. Approximately 50% of women take less than 80% of the prescribed dosage^28^ and up to 50% discontinue their treatment by the fifth year of prescription^29,30^. This pattern of poor adherence and early discontinuation of hormone therapy treatment are associated with a higher rate of relapse and mortality^6,31,32^. However, these side-effects are often underestimated and overseen in follow-up visits, that mainly focus on the risk of recurrence^10,33,34^. Indeed, one of the reasons why the impact of side-effects of oncology treatments is often overlooked is the non-inclusion of patient-reported outcomes (PROs) in the patient’s care protocols. As a consequence, some of these problems are underreported, making difficult its detection and treatment^10,34,35^.

Furthermore, these side-effects associated with AHT can severely affect the quality of life (QoL) and well-being of BC survivors^6,9,10,13,15,22,36–38^. Thus, for instance, they had a “disabling” effect^37^ with severe emotional implications, like social withdrawal^19^ and lack of professional confidence^16,22^, which in turn play a role in chronic pain, since it has been demonstrated that psychosocial factors play an important role in persistence and aggravation of pain^39,40^.

Nevertheless, and despite the relevance of the topic, data regarding the AHT impact on the QoL of BC survivors is scarce. Moreover, studies exploring this matter present some limitations, such as the absence of a control group (BC survivors without AHT) that allows for comparison and delimitation of the contribution of AHT to persistent symptoms and the recovery trajectory^41,42^. Also, a significant part of these previous studies focus on a relatively short period^21,25^, despite the fact that AHT is often prescribed for a long period.

Considering the impact of AHT side-effects on BC survivors, it becomes clear that further studies are advisable, in order to elucidate the role of the variables involved. One variable to consider on this regard is the time elapsed since end of primary treatment^10^. It has been suggested that given the different challenges involved, there should be considered three phases in the period following primary treatment^43^. A first phase takes place during the following year of primary treatment´s end, the re-entry phase. This first phase implies the transition from patient to survivor, the loss of the active treatment safety net and the frequent medical appointments^44,45^. After this first year of transition, two phases have been traditionally considered: the short-survival phase, which extends up to five after active treatment finalization; and the long survival-phase, which comprises from the fifth year after active treatment finalization onwards^45^. As aforementioned, each of these phases imply different adaptation challenges for the cancer survivor and, therefore, should be considered when aiming to facilitate and enhance such adaptation^44^.

For all of the above, the aim of the present study was to explore the modulatory role of AHT on health-related quality of life, as well as on subjective well-being and prevalence of distress in BC survivors, using a control group without AHT for comparison, and taking into consideration the survival phase. To that end, participant-reported outcomes (PROs) were used, including a quality-of-life assessment instrument specially developed and validated for cancer survivors. Also, three phases of survival were considered: re-entry, short, and long survivorship.

## Method

### Participants and procedure

A total of 733 BC survivors were approached in different medical institutions and Spanish cancer patient associations. The study was approved by the Research Ethics Committee of the Valencian Institute of Oncology Foundation FIVO and the Institute for Health Research INCLIVA. Inclusion criteria were: (a) previous diagnosis of BC and no signs of cancer at present, (b) finalization of the primary treatment with curative intent (surgery, chemotherapy and radiotherapy) at least one month before time of assessment, and (c) adequate command of the Spanish language. The final sample was composed of 616 women (84%) who provided informed consent and completed the questionnaires.

The mean age of the participants was 55.2 years (SD = 9.7) (range = 27–85). Most of them were married or living with a partner (72%) and had completed at least primary education (95.7%). Regarding employment status, 35.8% were working, 31.8% were retired or on sick leave, 16.1% were engaged in housework, and 13.7% were unemployed (Table 1). The most frequently treatment strategy received included surgery, chemotherapy and radiotherapy (61.0%), and almost two thirds of the women (63.2%) were given hormone therapy. About one in four participants (23.1%) had completed their primary treatment within the previous 12 months (Re-entry subgroup, RE), 33.6% had completed their primary treatment more than 5 years ago (Long Survival subgroup, LS), and 43.3% had completed their primary treatment more than 12 months ago but had not yet reached 5 years since finalization (Short Survival subgroup, SS).

**Table 1:** Descriptive of sociodemographic and disease-related variables (N = 616).

Variable	n (%)	With AHT 388 (63.0)	Without AHT 228 (36.0)	Chi^2^
**Age**				
≤ 45	97 (15.6)	63 (16.2)	34 (15.0)	
46–55	226 (36.3)	160 (41.0)	66 (29.2)	
56–65	204 (32.7)	114 (29.2)	90 (39.8)	11*
≥ 66	89 (14.3)	53 (13.6)	36 (15.9)	
*M* = 55.16; *SD* = 9.72; range = 27–85				
**Relationship status**				
Married/lived with partner	446 (72.5)	283 (73.5)	159 (71.3)	
Single/divorced/widowed	169 (27.5)	102 (26.5)	64 (28.7)	0.4
**Educational level**				
Without studies	25 (4.2)	8 (2.1)	17 (7.7)	
Primary education	238 (39.5)	149 (39.6)	87 (39.5)	
Secondary education	137 (22.8)	98 (26.1)	37 (16.8)	16.2**
University studies	202 (33.6)	121 (32.2)	79 (35.9)	
**Employment status**				
Active	219 (35.7)	151 (38.9)	67 (30.6)	
Unemployed	83 (13.5)	48 (12.4)	34 (15.5)	
Leave	120 (19.6)	77 (19.8)	41 (18.7)	
Retired	77 (12.6)	45 (11.6)	31 (14.2)	6.30
Housekeeper	98 (16.0)	59 (15.2)	38 (17.4)	
Other	16 (2.6)	8 (2.1)	8 (3.7)	
**Primary medical treatment**				
Surgery	34 (5.5)	21 (5.4)	13 (5.8)	
Surgery and radiotherapy	133 (21.6)	98 (25.2)	35 (15.5)	
Surgery and chemotherapy	49 (8.0)	19 (4.9)	30 (13.3)	
Surgery, chemotherapy and radiotherapy	376 (61.0)	238 (61.2)	137 (60.6)	19.8***
Others	24 (3.9)	13 (3.3)	11 (4.9)	

AHT, adjuvant hormone therapy.

* = p ≤ 0.05, ** = p ≤ 0.01, *** = p ≤ 0.001.

### Instruments

#### Quality of life

The Quality of Life (QoL) in Adult Cancer Survivors Scale (QLACS)^46^ is composed of 47 items that ask the subject about different aspects of his or her health-related quality of life during the previous month. It provides scores in twelve domains: seven considered generic (negative feelings, positive feelings, cognitive problems, pain, fatigue, social avoidance, and sexual problems) and five specifics to cancer (economic problems, family distress, concern about appearance, distress over recurrence, and cancer benefits) and also allows obtaining a total score from all of them, except for benefits^47^. Each domain is evaluated by 4 items (except family distress, which is assessed by only three and so the resulting score is multiplied by 1.33 in order to be able to be compared with the score in the rest of the domains) scored on a seven-point Likert scale. Higher scores indicate lower health-related quality of life (except in the domains of positive feelings and cancer benefits). We used the Spanish version of the QLACS^48^ which has shown satisfactory psychometric properties^47,48^. The internal consistency values obtained in this study were satisfactory (Cronbach's αTotal = 0.95; Cronbach's αsubscales range = 0.78–0.90).

#### Emotional distress

The Brief Symptom Inventory-18 (BSI-18)^49^ is a self-report instrument comprising 18 items rated on a five-point Likert scale that inquire about how the respondent has been feeling during the previous week. The instrument provides scores on three symptom subscales (somatization, depression, anxiety) and a total score (Global Severity Index, GSI). Moreover, the BSI-18 allows cases of clinical distress to be identified using normative data proposed by Derogatis^50^: the scores are transformed into T-scores (T ≥ 63 in the GSI or in at least two subscales). The Spanish version of the BSI-18 has shown satisfactory psychometric properties in the cancer population^51,52^. In the present study, the BSI-18 showed satisfactory internal consistency (Cronbach's α = 0.93); although it was used exclusively to determine the number of cases with clinically significant distress.

#### Subjective well-being

The Subjective Happiness Scale (SHS)^53^ (Spanish version by Extremera et al.^54^ is used as a subjective well-being indicator that is defined from the perspective of the person and includes multiple components (i.e., life satisfaction, positive and negative affect). It contains four self-report items, rated on a 7-point Likert scale. The internal consistency in the present study was satisfactory (Cronbach's α_Total_ = 0.84).

### Statistical analysis

Descriptive statistics were calculated to summarize the data for sociodemographic, cancer-related and psychosocial variables. A multivariate analysis of variance (MANOVA) was applied for the different domains of health-related QoL with AHT and survival phase as independent variables. Univariate F follow-up tests were conducted within the multivariate significant overall differences, and significant results on the univariate tests were followed with Bonferroni's comparisons between all possible pairs of means. Univariate F tests with Bonferroni's comparisons and Chi-square tests (depending on the variable considered) were also performed for the exploration of possible differences between the groups with and without AHT and the survival phases in total QoL, Benefits, Subjective Well-being and prevalence of Emotional Distress. The level of statistical significance used was p ≤ 0.05. All analyses were performed using the IBM SPSS statistical package, version 22.0.

### Ethics approval

All procedures performed in studies involving human participants were in accordance with the ethical standards of the institutional and/or national research committee and with the 1964 Helsinki Declaration and its later amendments or comparable ethical standards.

### Consent to participate

Informed consent was obtained from all individual participants included in the study.

## Results

Descriptive data of the sociodemographic and treatment variables and comparison between the subgroups with AHT and without AHT are shown in Table 1. Compared with the group without AHT, participants from the group with AHT were younger (p < 0.05), presented higher level of education (p > 0.01) and a smaller percentage of chemotherapy treatment (p > 0.001).

The results of Multivariate Analysis (MANOVA) in relation to health-related QoL showed no significant main effect of AHT (Wilks' lambda = 0.97, F(11, 608) = 1.67, p = . 078). However, it showed a significant main effect of survival phase (Wilks' lambda = 0.92, F(22, 1192) = 2.21, p = 0.001), as well as a significant effect of the interaction between both variables (survival phase x AHT) (Wilks' lambda = 0.95, F(22, 1192) = 1.56, p = 0.048).

Univariate F values are shown in Tables 2 and 3. Regarding the survival phase (Table 2), the results indicate an improvement in negative feelings (p < 0.01), positive feelings (p < 0.01), fatigue (p < 0.01) and social avoidance (p < 0.05) when considering the participants as a whole. At longer elapsed time, BC survivors also show an improvement in total QoL (p < 0.05), perceived benefits (p < 0.01) and subjective well-being (p < 0.05), as well as a decrease in the prevalence of emotional distress (p < 0.05).

**Table 2:** ANOVA, post-hoc tests and Chi^2^ for total sample by hormone therapy and survival phase phase.

	RE (N = 142) Mean (SD)	SS (N = 267) Mean (SD)	LS (N = 207) Mean (SD)	F
Negative feelings	13.9 (5.1)^c^	13.6 (5.5)^c^	12.3 (4.5)^a,b^	5.0**
Positive feelings	18.7 (6.1)^c^	19.8 (6.1)	20.7 (5.6)^a^	4.8**
Cognitive problems	12.8 (6.1)	12.5 (6.0)	12.0 (5.1)	0.8
Pain	13.0 (5.9)	12.8 (6.9)	12.0 (5.9)	1.5
Fatigue	14.8 (5.1)^c^	14.1 (6.1)	12.8 (5.6)^a^	5.4**
Social avoidance	9.5 (6.0)^c^	8.7 (5.8)	7.8 (4.4)^a^	4.1*
Sexual problems	13.7 (7.2)	13.4 (7.0)	12.4 (6.1)	1.9
Financial problems	7.1 (5.0)	7.8 (5.9)	6.8 (5.0)	2.1
Family distress	18.8 (7.7)	17.2 (8.0)	18.6 (7.6)	2.9
Appearance	13.0 (6.7)	11.7 (6.7)	11.4 (6.9)	2.7
Distress recurrence	15.2 (6.7)	14.8 (6.8)	15.1 (7.1)	0.1
Total score	145.0 (44.3)^c^	138.8 (49.3)	132.6 (40.2)^a^	3.2*
Benefits	18.0 (6.8)^c^	18.5 (6.9)^c^	20.0 (6.2)^a,b^	4.9**
Subjective well-being	20.0 (5.4)^c^	20.2 (5.4)^c^	21.4 (4.7)^a,b^	4.2*
Emotional distress	56 (37.6)	74 (27.7)	53 (25.7)	6.5*

RE, re-entry subgroup; SS, short survival subgroup; LS, long survivor subgroup.

* = p ≤ 0.05, ** = p ≤ 0.01, *** = p ≤ 0.001.

^a^Significant differences with re-entry phase,^b^significant differences with short survival phase,^c^significant differences with long survival phase. The family distress subscale has been weighted in order to equalize the rank with the rest of the subscales.

**Table 3:** ANOVA, post hoc tests and Chi2 for subgroups with and without hormone therapy by survival phase.

	Subgroup with AHT (N = 388)				Subgroup without AHT (N = 228)			
	RE (N = 104) Mean (SD)	SS (N = 184) Mean (SD)	LS (N = 100) Mean (SD)	F	RE (N = 38) Mean (SD)	SS (N = 83) Mean (SD)	LS (N = 107) Mean (SD)	F
Negative feelings	13.5 (5.3)	13.4 (5.4)	12.9 (4.7)	0.4	14.9 (4.7)	14.2 (5.8)^c^	11.7 (4.2)^b^	8.5***
Positive feelings	18.8 (6.2)^c^	20.2 (5.9)	20.9 (5.8)^a^	3.2*	18.3 (5.7)	19.1 (6.3)	20.6 (5.3)	3.0
Cognitive problems	12.2 (6.2)	12.7 (5.9)	11.9 (5.1)	0.7	14.4 (5.6)	12.0 (6.3)	12.0 (5.1)	2.7
Pain	12.7 (5.7)	12.7 (6.7)	12.5 (6.1)	0.0	13.8 (6.4)	13.3 (7.3)^c^	11.3 (5.5)^b^	3.2*
Fatigue	14.2 (4.9)	14.1 (6.2)	12.7 (5.6)	2.5	16.2 (5.5)^c^	14.0 (5.9)	12.9 (5.5)^a^	4.6*
Social avoidance	9.4 (5.9)	8.4 (5.7)	8.2 (4.8)	1.4	9.6 (6.1)	9.3 (5.9)	7.5 (3.9)	3.9*
Sexual problems	13.5 (7.3)	13.0 (6.8)^c^	13.1 (6.4)	0.2	14.3 (6.9)	14.3 (7.2)^c^	11.8 (5.7)^b^	4.3*
Financial problems	6.7 (4.4)	7.5 (5.6)	6.8 (5.4)	1.2	8.1 (6.3)	8.1 (6.2)	6.7 (4.5)	1.9
Family distress	18.4 (7.9)	17.0 (7.8)	18.1 (7.8)	1.2	20.1 (6.9)	17.4 (8.4)	19.1 (7.5)	1.9
Appearance	12.5 (6.7)	11.2 (6.6)	10.4 (6.6)	2.5	14.4 (6.7)	12.8 (6.8)	12.1 (6.9)	1.5
Distress recurrence	15.4 (6.9)	14.7 (6.7)	15.0 (7.3)	0.4	14.0 (6.1)	15.3 (7.0)	15.2 (6.9)	0.5
Total score	141.6 (45.2)	136.7 (46.9)	132.7 (41.7)	0.4	153.5 (42.0)^c^	143.6 (54.4)	131.6 (38.3)^a^	3.7*
Benefits	17.2 (7.1)^c^	18.2 (7.2)	19.6 (6.5)^a^	3.2*	19.6 (6.0)	19.0 (6.2)	20.3 (6.0)	1.1
Subjective well-being	20.2 (5.4)	20.6 (5.1)	21.5 (4.9)	1.7	19.4 (5.3)	19.5 (6.0)^c^	21.4 (4.5)^b^	3.8*
Emotional distress	37 (34.9)	46 (25.4)	27 (27.3)	3.1	19 (46.3)	28 (32.9)	25 (23.6)	7.4*

AHT, adjuvant hormone therapy; RE, re-entry subgroup; SS, short survival subgroup; LS, long survivor subgroup.

* = p ≤ 0.05, ** = p ≤ 0.01, *** = p ≤ 0.001.

^a^Significant differences with re-entry phase,^b^significant differences with short survival phase,^c^significant differences with long survival phase. The family distress subscale has been weighted in order to equalize the rank with the rest of the subscales.

Analysis of the interaction effect showed (Table 3) that in the subgroup with AHT, health-related QoL improved significantly only in the domain of positive feelings (p < 0.05), whereas in the subgroup without AHT there was an improvement in several domains: negative feelings (p < 0.001), pain, fatigue, social avoidance and sexual problems (p < 0.05 in all these cases). Furthermore, these differences resulted in an improvement in total QoL in the subgroup without AHT (p < 0.05). The results also showed that the participants with AHT only increased their perception of benefits (p < 0.05), while in the group without AHT, subjective well-being increased (p < 0.05) and the prevalence of emotional distress decreased (p < 0.05).

Finally, the differences were predominantly to be found between the long survival phase and the re-entry or short survival phases.

## Discussion

The aim of this work was to explore the modulating role of AHT in the QoL, subjective well-being and emotional distress prevalence of BC survivors.

First of all, regarding the comparison of the sociodemographic variables between the subgroups with- and without- AHT, the first was slightly younger, more educated and with a lower percentage chemotherapy as primary treatment than the later. While these differences may affect the comparability of the subgroups on QoL, distress and well-being, the meaning of these differences regarding the main findings of the study vary according to the direction of the association found in previous research. Although a positive association has been found between age and QoL^55,56^; studies also indicate a negative association between QoL and chemotherapy treatment^57,58^ and, to a lesser extent, to educational level^56,59^. It cannot be concluded, therefore, that the profile of the group with AHT has biased the results regarding their worse QoL.

Furthermore, when the survival phase was not included in the analyses our results showed no effect of hormone therapy. It appears that the impact of primary treatment on QoL of BC survivors masks the specific effect of hormone therapy. In this respect, previous research^38,60^ have shown the negative impact of treatment combining radiotherapy and chemotherapy on the patient’s QoL (which is the case for most of the participants in this study).

When considering time since end of primary treatment, our results reflect significant impact of the cancer on QoL particularly marked in the re-entry phase, as well as an improvement experienced by survivors towards the long-term phase, which goes in line with previous research^47^. However, examination of interaction effect between the presence or absence of AHT and time since end of primary treatment showed that many of the changes observed through the survival phases were experienced exclusively by survivors without AHT.

Furthermore, the group with AHT only showed an increase in positive feelings and perceived benefits, while the rest of the improvements observed in the total sample were exclusively experienced by the subgroup without AHT. In other words, when AHT was not prescribed, longer time elapsed was associated with a decrease in emotional distress and an improvement in subjective well-being and overall QoL. This improvement in QoL was specifically reflected by a decrease in negative feelings, fatigue, social avoidance, pain and sexual problems (the latter two were not detected when analysing the total sample). Thus, it appears that AHT hinders the physical and emotional recovery of BC survivor presumably due to the numerous side-effects of medication and the burden that they suppose on everyday life. Since the differences were found between the re-entry and short survival phases, and the long survival one, it seems that there is a turning point around the fifth year after finalization of primary treatment, from which the survivors without AHT significantly improve in several areas and those with AHT do so to a significant lesser extent.

It is worth noting that, compared to the subgroup without AHT, survivors with AHT did not experienced improvement in three areas that are clearly associated with some of the most frequently reported side-effects of AHT, namely: fatigue, pain (associated with musculoskeletal symptoms such as myalgias and arthralgias) and sexual problems^11,12,15,22,35,36,61^. Moreover, the decrease observed in this symptomatology in the subgroup without AHT was associated with a reduction of negative affectivity (even when assessed as prevalence of emotional distress) and social avoidance, and an increase in subjective well-being. However, none of these improvements were found in the AHT subgroup.

At the same time, the increased perception of benefits in the AHT subgroup can be understood as a cognitive emotion regulation strategy of a functional nature that involves a reappraisal by generating a positive interpretation of the stressful situation^62,63^. The reappraisal of their situation enable survivors undergoing hormone therapy to also retain a certain sense of control by establishing a secondary control^64^. The relevance and persistence of symptoms seems, however, to considerably limit the effectiveness of this strategy: only one of the two indicators of positive affectivity and neither of the two indicators of negative affectivity showed improvement in this subgroup.

The mainly focus on the risk of recurrence^33,34^ in follow-up visits and the fact that AHT is administered on an outpatient basis through the ingestion of a simple pill may be leading to underestimate and, therefore, to neglect the important negative side-effects of this therapy in the recovery of women who have suffered from BC. In fact, many endocrine-related side-effects go under-recognized, underreported, and undertreated^10,33–35^.

The burden of these unaddressed symptoms on BC survivors is further underscored by (1) the long duration of treatment (minimum 5 years) and (2) the high prevalence (roughly 75%) of hormone receptor-positive BC^65^ and it should not be underestimated. Quite on the contrary, AHT side-effects should be routinely assessed by health care providers in order to gain a more accurate knowledge that allows a significant improvement in the QoL of the BC survivor undergoing AHT^66^. In fact, data suggest that a reduction in symptom burden may come from improved physician-survivor communication^67^. Moreover, unlike sociodemographic and clinical factors that are not amenable to change, side effects are suggested interventions targets^13^. This would certainly be feasible, since current research suggests that evidence-based strategies (pharmacological and non-pharmacological) are currently available in order to manage specific side-effects associated with AHT, such as fatigue, sexual dysfunction, and musculoskeletal symptoms.

In addition, it is expected that the improvement in QoL throughout the different survival phases will have a significant impact on the adherence and maintenance of AHT and, consequently, in the likelihood of survival. Symptom’s management is a key element of survivorship care that must be considered in order to achieve a balance between benefits and loses that ensures optimal treatment outcomes without neglecting issues related to treatment adherence, tolerability of side-effects and associated quality of life^61,66^.

Despite its meaningful contributions, this study is not without limitations. The cross-sectional design prevents the establishment of causal relationships. In addition, the small size of some of the subgroups established for comparative purposes advises to be cautious about the reliability of some of the results. Nevertheless, we consider that our results are conservative in detecting differences through the survival phases regarding the evolution of QoL, subjective well-being and emotional distress of survivors with and without AHT. This is so, given that the smallest subgroups corresponded to the survivors without hormone therapy. A further limitation of our study is the absence data regarding the menopausal status of the participants and the different types of AHT agents used (mostly tamoxifen in premenopausal and AIs in postmenopausal women). Information on this regard would be useful considering the differences in QoL deterioration associated to each type of hormonal medication^25^. Also, the consideration of other variables that could not be contemplated in this study would also be advisable, such as aggressiveness of tumours (e.g., stage, HER2-positive, triple-negative tumours), that could affect women's self-reported QoL. However, disease stage is not associated with hormone therapy administration and patients with triple negative tumours do not receive hormone therapy and only those patients with HER2 tumours that also express hormone receptors will receive hormone therapy^68–72^. Thus, since these tumours will necessarily be more represented in the subgroup without hormone therapy, the worse QoL of hormone-treated participants does not seem likely to be the result of the presence of more aggressive tumours.

Future studies with larger samples, longitudinal designs and specific information about the aforementioned aspects are needed in order to deepen in the impact of hormone therapy on the quality of life, subjective well-being and emotional distress of BC survivors. The present study highlights the relevance of this matter and the need to address it from the research, the clinical and the regulatory policies fields and it especially suggests that further research on this regard is highly needed in order to evaluate the pros and cons of a maintenance treatment strategy, and to tailor it aiming to facilitate treatment adherence and safeguard the well-being and quality of life of BC survivors.

## Conclusion

In short, our study explored the modulatory role of AHT on the QoL of a large sample of BC survivors, taking into account three survival phases (re-entry, short and long survival), and using a Quality of Life instrument created specifically for the population of cancer survivors. It also does so comparing survivors with AHT with a control group of survivors without AHT. Our results highlight how the survivor’s trajectory is different whereas they have received AHT or not. Also, is seems that AHT hinders the physical and emotional recovery of BC survivors even in the long survival phase.

## Data Availability

The datasets generated during and/or analysed during the current study are available from the corresponding author on reasonable request.

## References

[1] WHO.* Breast Cancer: Scope of the Problem*. https://www.who.int/news-room/fact-sheets/detail/breast-cancer (accessed 08 March 2022).

[2] Miller KD, Nogueira L, Mariotto AB (2019). Cancer treatment and survivorship statistics. CA Cancer J. Clin..

[3] Waks GA, Winer EP (2019). Breast cancer treatment: A review. JAMA.

[4] Fabian CJ (2007). The what, why and how of aromatase inhibitors: Hormonal agents for treatment and prevention of breast cancer. Int. J. Clin. Pract..

[5] Early Breast Cancer Trialists’ Collaborative Group (2015). Aromatase inhibitors versus tamoxifen in early breast cancer: Patient-level meta-analysis of the randomised trials. Lancet.

[6] Peddie N, Agnew S, Crawford M, Dixon D, MacPherson I, Fleming L (2021). The impact of medication side-effects on adherence and persistence to hormone therapy in breast cancer survivors: A qualitative systematic review and thematic synthesis. The Breast.

[7] Sheng JY, Blackford AL, Bardia A, Venkat R, Rosson G, Giles J (2019). Prospective evaluation of finger two-point discrimination and carpal tunnel syndrome among women with breast cancer receiving adjuvant aromatase inhibitor therapy. Breast Cancer Res. Treat..

[8] Leysen L, Beckwee D, Nijs J, Pas R, Bilterys T, Vermeir S, Adriaenssens N (2017). Risk factors of pain in breast cancer survivors: A systematic review and meta-analysis. Support. Care Cancer.

[9] Wells KJ, Pan TM, Vázquez-Otero C, Ung D, Ustjanauskas AE, Muñoz D, Laronga C, Roetzheim RG, Goldenstein M, Carrizosa C (2016). Barriers and facilitators to endocrine therapy adherence among underserved hormone-receptor-positive breast cancer survivors: A qualitative study. Support. Care Cancer.

[10] Heins MJ, de Ligt KM, Verloop J, Siesling S, Korevaar JC, Berendsen A (2022). Adverse health effects after breast cancer up to 14 years after diagnosis. The Breast.

[11] Amir E, Seruga B, Niraula S, Carlsson L, Ocaña A (2011). Toxicity of adjuvant endocrine therapy in postmenopausal breast cancer patients: A systematic review and meta-analysis. J. Natl. Cancer Inst..

[12] Howard-Anderson J, Ganz PA, Bower JE, Stanton AL (2012). Quality of life, fertility concerns, and behavioral health outcomes in younger breast cancer survivors: A systematic review. J. Natl. Cancer Inst..

[13] Moon Z, Moss-Morris R, Hunter MS, Hughes LD (2017). Understanding tamoxifen adherence in women with breast cancer: A qualitative study. Br. J. Health. Psychol..

[14] Cahir C, Guinan E, Dombrowski SU, Sharp L, Bennett K (2015). Identifying the determinants of adjuvant hormonal therapy medication taking behaviour in women with stages I-III breast cancer: A systematic review and meta-analysis. Patient Educ. Couns..

[15] Nimbi FM, Magno S, Agostini L, Di-Micco A, Maggiore C, De-Cesaris BM, Tambelli R (2022). Sexuality in breast cancer survivors: Sexual experiences, emotions, and cognitions in a group of women under hormonal therapy. Breast Cancer.

[16] van Londen GJ, Donovan HS, Beckjord EB, Cardy AL, Bovbjerg DH, Davidson NE, Morse JQ, Switzer GE, Verdonck-de Leeuw IM, Dew MA (2014). Perspectives of postmenopausal breast cancer survivors on adjuvant endocrine therapy-related symptoms. Oncol. Nurs. Forum NIH Public Access.

[17] Bluethmann SM, Murphy CC, Tiro JA, Mollica MA, Vernon SW, Bartholomew LK (2017). Deconstructing decisions to initiate, maintain, or discontinue adjuvant endocrine therapy in breast cancer survivors: A mixed-methods study. Oncol. Nurs. Forum NIH Public Access.

[18] Humphries B, Collins S, Guillaumie L, Lemieux J, Dionne A, Provencher L, Moisan J, Lauzier S (2018). Women’s beliefs on early adherence to adjuvant endocrine therapy for breast cancer: A theory-based qualitative study to guide the development of community pharmacist interventions. Pharmacy..

[19] Lambert LK, Balneaves LG, Howard A, Chia SK, Gotay CC (2018). Understanding adjuvant endocrine therapy persistence in breast Cancer survivors. BMC Cancer.

[20] Lovelace DL, McDaniel LR, Golden D (2019). Long-term effects of breast cancer surgery, treatment, and survivor care. J. Midwifery Womens Health.

[21] Ganz PA, Goodwin PJ. Breast cancer survivorship: Where are we today? In *Improving Outcomes for Breast Cancer Survivors* 1–8 (Springer, Cham, 2015).

[22] Brauer ER, Ganz PA, Pieters HC (2016). “Winging It”: How older breast cancer survivors persist with aromatase inhibitor treatment. J. Oncol. Pract..

[23] Friese C, Pini T, Li Y, Abrahame P, Graff J, Hamilton A (2013). Adjuvant endocrine therapy initiation and persistence in a diverse sample of patients with breast cancer. Breast Cancer Res. Treat..

[24] Kimmick G, Edmond S, Bosworth H, Peppercorn J, Marcom P, Blackwell K (2015). Medication taking behaviors among breast cancer patients on adjuvant endocrine therapy. Breast.

[25] Ferreira AR, Di Meglio A, Pistilli B (2019). Differential impact of endocrine therapy and chemotherapy on quality of life of breast cancer survivors: A prospective patient-reported outcomes analysis. Ann. Oncol..

[26] Beckwée D, Leysen L, Meuwis K, Adriaenssens N (2017). Prevalence of aromatase inhibitor-induced arthralgia in breast cancer: A systematic review and meta-analysis. Support. Care Cancer.

[27] Hershman DL, Loprinzi C, Schneider BP (2015). Symptoms: Aromatase Inhibitor Induced Arthralgias. Improving Outcomes for Breast Cancer Survivors.

[28] Moon Z, Moss-Morris R, Hunter MS, Norton S, Hughes LD (2019). Nonadherence to tamoxifen in breast cancer survivors: A 12 month longitudinal analysis. Health Psychol..

[29] Hadji P, Ziller V, Kyvernitakis J, Bauer M, Haas G, Schmidt N, Kostev K (2013). Persistence in patients with breast cancer treated with tamoxifen or aromatase inhibitors: A retrospective database analysis. Breast Cancer Res. Treat..

[30] Makubate B, Donnan PT, Dewar JA, Thompson AM, McGowan C (2013). Cohort study of adherence to adjuvant endocrine therapy, breast cancer recurrence and mortality. Br. J. Cancer..

[31] Dezentje VO, van Blijderveen NJ, Gelderblom H, Putter H, van Herk-Sukel MP, Casparie MK (2010). Effect of concomitant CYP2D6 inhibitor use and tamoxifen adherence on breast cancer recurrence in early-stage breast cancer. J Clin Oncol..

[32] Brito C, Portela MC, Vasconcellos MT (2014). Factors associated to persistence with hormonal therapy in women with breast cancer. Rev. Saude Publ..

[33] Ribi K, Luo W, Bernhard J (2016). Adjuvant tamoxifen plus ovarian function suppression versus tamoxifen alone in premenopausal women with early breast cancer: Patient-reported outcomes in the suppression of ovarian function trial. J. Clin. Oncol..

[34] de-Ligt KM, van-Egdom LSE, Koppert LB, Siesling S, van-Til JA (2019). Opportunities for personalised follow-up care among patients with breast cancer: A scoping review to identify preference-sensitive decisions. Eur. J. Cancer Care (Engl.).

[35] Fallowfield L, Jenkins V (2015). Psychosocial/survivorship issues in breast cancer: Are we doing better?. J. Natl. Cancer Inst..

[36] Ahlstedt Karlsson S, Wallengren C, Olofsson Bagge R, Henoch I (2019). “It is not just any pill”—women’s experiences of endocrine therapy after breast cancer surgery. Eur. J. Cancer Care.

[37] Iacorossi L, Gambalunga F, Fabi A, Giannarelli D, Marchetti A, Piredda M, De-Marinis MG (2018). Adherence to oral administration of endocrine treatment in patients with breast cancer: A qualitative study. Cancer Nurs..

[38] Andreu Y, Martínez P, Soto-Rubio A, Pérez-Marín M, Cervantes A, Arribas L (2022). Quality of life in cancer survivorship: Sociodemographic and disease-related moderators. Eur. J. Cancer Care.

[39] Turk DC, Wilson HD (2010). Fear of pain as a prognostic factor in chronic pain: Conceptual models, assessment, and treatment implications. Curr. Pain Headache Rep..

[40] Rashiq S, Dick BD (2014). Post-surgical pain syndromes: A review for the non-pain specialist. Can. J. Anesthesia.

[41] Fallowfield L (2007). Quality of life issues in relation to the aromatase inhibitor. J. Steroid Biochem. Mol. Biol..

[42] Fallowfield LJ, Bliss JM, Porter LS, Price MH, Snowdon CF, Jones SE, Coombes RC, Hall E (2006). Quality of life in the intergroup exemestane study: A randomized trial of exemestane versus continued tamoxifen after 2 to 3 years of tamoxifen in postmenopausal women with primary breast cancer. J. Clin. Oncol..

[43] Stanton AL, Rowland JH, Ganz PA (2015). Life after diagnosis and treatment of cancer in adulthood: Contributions from psychosocial oncology research. Am. Psychol..

[44] Chao YH, Wang SY, Sheu SJ (2020). Integrative review of breast cancer survivors’ transition experience and transitional care: Dialog with transition theory perspectives. Breast Cancer.

[45] Williamson TJ, Stanton AL (2018). Adjustment to Life as a Cancer Survivor. Handbook of Cancer Survivorship.

[46] Avis NE, Smith KW, McGraw S, Smith RG, Petronis VM, Carver CS (2005). Assessing quality of life in adult cancer survivors (QLACS). Qual. Life Res..

[47] Andreu Y, Conchado A, Martinez P, Martinez MT, Moreno P, Arribas L (2021). Possible substantive improvements in the structure of the Quality of Life in Adult Cancer Survivors (QLACS) scale? A study based on its Spanish version. Qual. Life Res..

[48] Escobar A, Trujillo-Martín MD, Rueda A, Pérez-Ruiz E, Avis NE, Bilbao A (2015). Cross-cultural adaptation, reliability and validity of the Spanish version of the Quality of Life in Adult Cancer Survivors (QLACS) questionnaire: Application in a sample of short-term survivors. Health Qual. Life Outcomes.

[49] Derogatis LR. *Brief Symptom Inventory 18 (BSI-18) Manual*. Minnetonka, MN: NCS Assessments (2001)

[50] Derogatis, L.R. Brief Symptom Inventory 18. (2001). https://www.pearsonclinical.com/products/100000638/brief-symptom-inventory-18-bsi-18.html#tab-details.

[51] Galdón MJ, Durá E, Andreu Y, Ferrando M, Murgui S, Pérez S, Ibañez E (2008). Psychometric properties of the Brief Symptom Inventory-18 in a Spanish breast cancer sample. J. Psychosom. Res..

[52] Martínez-López P, Conchado-Peiró A, Andreu-Vaillo Y, Galdón-Garrido MJ (2019). Psychometric properties of the Brief Symptom Inventory-18 in a heterogeneous sample of adult cancer patients. Rev. Latinoamericana Psicol..

[53] Lyubomirsky S, Lepper HS (1999). A measure of subjective happiness: Preliminary reliability and construct validation. Soc. Indic. Res..

[54] Extremera N, Fernández-Berrocal P (2014). The Subjective Happiness Scale: Translation and preliminary psychometric evaluation of a Spanish version. Soc. Indic. Res..

[55] Azam S, Eriksson M, Sjölander A, Gabrielson M, Hellgren R, Czene K (2021). Mammographic microcalcifications and risk of breast cancer. Br. J. Cancer.

[56] Maisarah S, Chan HK, Ah MR (2021). Delayed presentation of colorectal cancer patients in Northern Malaysia: 11-Year prevalence, associated factors and impact on survival. Int. J. Public Health Clin. Sci..

[57] Akel R, El Darsa H, Anouti B, Mukherji D, Temraz S, Raslan R, Tfayli A, Assi H (2017). Anxiety, depression and quality of life in breast cancer patients in the levant. Asian Pac. J. Cancer Prevent..

[58] Brenkman HJF, Tegels JJW, Ruurda JP, Luyer MDP, Kouwenhoven EA, Draaisma WA (2018). Factors influencing health-related quality of life after gastrectomy for cancer. Gastric Cancer.

[59] Hechtner M, Krause M, König J, Appold S, Hornemann B, Singer S, Baumann M (2018). Long-term quality of life in inoperable non-small cell lung cancer patients treated with conventionally fractionated compared to hyperfractionated accelerated radiotherapy—results of the randomized CHARTWEL trial. Radiother. Oncol..

[60] Peters E, Schulz LM, Reuss-Borst M (2016). Quality of life after cancer—how the extent of impairment is influenced by patient characteristics. BMC Cancer.

[61] Franzoi MA, Agostinetto E, Perachino M, Del Mastro L, de Azambuja E, Vaz-Luis I, Partridge AH, Lambertini M (2021). Evidence-based approaches for the management of side-effects of adjuvant endocrine therapy in patients with breast cancer. Lancet Oncol..

[62] Gross JJ (1998). Antecedent-and response-focused emotion regulation: Divergent consequences for experience, expression, and physiology. J. Personal. Soc. Psychol..

[63] Rottenberg J, Ray RR, Gross JJ, Coan JA, Allen JJB (2007). Emotion elicitation using films. The Handbook of Emotion Elicitation and Assessment.

[64] Rothbaum F, Weisz JR, Snyder SS (1982). Changing the world and changing the self: A two-process model of perceived control. J. Pers. Soc. Psychol..

[65] Felder TM, Heiney SP, Hebert JR, Friedman DB, Elk R, Franco R, Gansauer L, Christensen B, Ford ME (2020). Improving adherence to adjuvant hormonal therapy among disadvantaged women diagnosed with breast cancer in South Carolina: Proposal for a multimethod study. JMIR Res. Protoc..

[66] Zeng Y, Huang M, Cheng AS, Zhou Y, So WK (2014). Meta-analysis of the effects of exercise intervention on quality of life in breast cancer survivors. Breast Cancer.

[67] Street RL, Makoul G, Arora NK, Epstein RM (2009). How does communication heal? Pathways linking clinician–patient communication to health outcomes. Patient Educ. Counsel..

[68] Azim HA, Michiels S, Bedard PL, Singhal SK, Criscitiello C, Ignatiadis M, Haibe-Kains B, Piccart MJ, Sotiriou C, Loi S (2012). Elucidating prognosis and biology of breast cancer arising in young women using gene expression profiling prognosis and biology of breast cancer in young women. Clin. Cancer Res..

[69] Vriens IJH, De Bie AJR, Aarts MJB, de Boer M, van Hellemond IEG, Roijen JHE, van Golde RJT, Voogd AC, Tjan-Heijnen VCG (2017). The correlation of age with chemotherapy-induced ovarian function failure in breast cancer patients. Oncotarget.

[70] Partridge AH, Ruddy KJ, Gelber S, Schapira L, Abusief M, Meyer M, Ginsburg E (2010). Ovarian reserve in women who remain premenopausal after chemotherapy for early stage breast cancer. Fertil. Steril..

[71] Prat A, Baselga J (2008). The role of hormonal therapy in the management of hormonal-receptor-positive breast cancer with co-expression of HER2. Nat. Clin. Pract. Oncol..

[72] Wahba HA, El-Hadaad HA (2015). Current approaches in treatment of triple-negative breast cancer. Cancer Biol. Med..

